# Improving Adherence to Web-Based and Mobile Technologies for People With Psychosis: Systematic Review of New Potential Predictors of Adherence

**DOI:** 10.2196/mhealth.7088

**Published:** 2017-07-20

**Authors:** Clare Killikelly, Zhimin He, Clare Reeder, Til Wykes

**Affiliations:** ^1^ Institute of Psychiatry, Psychology & Neuroscience Department of Psychology King's College London London United Kingdom; ^2^ Division of Psychopathology and Clinical Intervention Department of Psychology University of Zurich Zurich Switzerland; ^3^ Department of Psychology Aberystwyth University Ceredigion United Kingdom; ^4^ South London and Maudsley NHS Foundation Trust London United Kingdom

**Keywords:** patient compliance, schizophrenia spectrum and other psychotic disorders, mobile phone, mHealth

## Abstract

**Background:**

Despite the boom in new technologically based interventions for people with psychosis, recent studies suggest medium to low rates of adherence to these types of interventions. The benefits will be limited if only a minority of service users adhere and engage; if specific predictors of adherence can be identified then technologies can be adapted to increase the service user benefits.

**Objective:**

The study aimed to present a systematic review of rates of adherence, dropout, and approaches to analyzing adherence to newly developed mobile and Web-based interventions for people with psychosis. Specific predictors of adherence were also explored.

**Methods:**

Using keywords (Internet or online or Web-based or website or mobile) AND (bipolar disorder or manic depression or manic depressive illness or manic-depressive psychosis or psychosis or schizophr* or psychotic), the following databases were searched: OVID including MedLine, EMBASE and PsychInfo, Pubmed and Web of Science. The objectives and inclusion criteria for suitable studies were defined following PICOS (population: people with psychosis; intervention: mobile or Internet-based technology; comparison group: no comparison group specified; outcomes: measures of adherence; study design: randomized controlled trials (RCT), feasibility studies, and observational studies) criteria. In addition to measurement and analysis of adherence, two theoretically proposed predictors of adherence were examined: (1) level of support from a clinician or researcher throughout the study, and (2) level of service user involvement in the app or intervention development. We provide a narrative synthesis of the findings and followed the preferred reporting items for systematic reviews and meta-analyses (PRISMA) guidelines for reporting systematic reviews.

**Results:**

Of the 20 studies that reported a measure of adherence and a rate of dropout, 5 of these conducted statistical analyses to determine predictors of dropout, 6 analyzed the effects of specific adherence predictors (eg, symptom severity or type of technological interface) on the effects of the intervention, 4 administered poststudy feedback questionnaires to assess continued use of the intervention, and 2 studies evaluated the effects of different types of interventions on adherence. Overall, the percentage of participants adhering to interventions ranged from 28-100% with a mean of 83%. Adherence was greater in studies with higher levels of social support and service user involvement in the development of the intervention. Studies of shorter duration also had higher rates of adherence.

**Conclusions:**

Adherence to mobile and Web-based interventions was robust across most studies. Although 2 studies found specific predictors of nonadherence (male gender and younger age), most did not specifically analyze predictors. The duration of the study may be an important predictor of adherence. Future studies should consider reporting a universal measure of adherence and aim to conduct complex analyses on predictors of adherence such as level of social presence and service user involvement.

## Introduction

E-mental health interventions, defined as “the use of information and communication technology to support or improve mental health care” [1,2], have been proposed as promising alternatives to traditional interventions. Proposed benefits include ease of use, accessibility, and the potential to be less stigmatizing [3-5]. This may be particularly appealing for service users with psychosis who tend to have high relapse rates yet limited access to psychological therapies [4,6]. Psychosis is a debilitating mental health disorder that includes symptoms such as hallucinations, delusions, disorganized thoughts, and speech, as well as diminished emotional expression and lack of volition [7]. Dropout and nonadherence rates for traditional psychological and psychopharmacological interventions are high for people with psychosis. “Dropout” is defined as noncompletion of the study protocol or the study assessments, and “adherence” is defined as the extent to which a participant experiences or engages with a mobile or Internet-based intervention [8]. Dropout rates of 25% for people with psychosis [9-11] and 30-57% for people with first episode psychosis (FEP) are commonly found [12]. Some have suggested that e-mental health technologies may provide a more acceptable therapy format than traditional face-to-face therapy [13]. Rates of adherence across different types of e-mental health interventions for people with psychosis have not been systematically examined.

A recent review of 12 studies highlighted that a specific examination of adherence, the extent to which a participant engages with an intervention, would be helpful for the field of e-mental health [14]. The study demonstrated that service users with psychosis varied in their engagement with the technological interventions; some showed regular or intermittent use and approximately 25-30% of participants did not engage or dropped out [14]. We seek to update this 2013 review for two main reasons. First, since 2013, there has been a dramatic increase in peer-reviewed publications examining Web-based or mobile technologies for a variety of mental health conditions. When reviewing the publication rate of e-mental health papers over the past 20 years, 57% were published in the last 5 years and the number of publications tripled between 2009-2014 [15]. Higgins and Green (2011) recommend that review updates should be carried out every 2 years, especially in a rapidly growing field [24]. Second, examining use and adherence to these new technologies is increasingly important as the benefits are limited if service users do not use them.

In order to obtain an overview of the rates of adherence, two types of adherence rates were collected: (1) mean percentage of the intervention completed and (2) percent of participants that complete the intervention [16]. Previous systematic reviews have developed four main approaches to examining adherence to mobile or Web-based interventions for treatment of depression and anxiety [8,16] (see Table 1 for an overview). The first is to examine factors that contribute to dropout from a study; for example, a comparison of baseline symptomology or demographic factors in participants who stay in the study and those who drop out. The second is to conduct statistical analyses, including correlational or regression analyses within a study to identify potential predictors of adherence. Specific service user factors (eg, demographics and clinical severity) and intervention factors (eg, week 1 vs week 2 of intervention) are most commonly explored. The third is to use questionnaires to retrospectively examine participants’ experiences of adherence and perspectives on continued use. The fourth approach is to experimentally manipulate factors within a study to impact upon adherence; for example, to compare different technological interfaces, frequency of use, or behavioral interventions.

**Table 1:** Four approaches to studying adherence.

Approach	Type of adherence data expected
Analysis of dropout data	Comparison of adherent and nonadherent service-user data including demographic, symptom, cognitive or other data; baseline assessment of between group differences
Within studies analyses to establish relationship between adherence and various factors	Within study correlational, regression or other analysis of service-user specific factors or intervention specific factors that may impact on the level of adherence to intervention or technology
Poststudy questionnaire on participants’ experience	Questionnaire data; qualitative or quantitative feedback on satisfaction, acceptability of study or intervention with specific questions on usability, helpfulness, and continued use
Experimental manipulation of factors impacting adherence	Comparison of interventions or interfaces that are specifically designed to impact on adherence

In addition to these four approaches to studying adherence, we evaluated two theoretically proposed predictors of adherence: (1) level of social presence or contact with a researcher, clinician or peer, and (2) servicer user involvement in the development of the intervention or app. The level of social presence or contact refers to the frequency and quality of clinician, researcher, or peer presence or contact throughout the intervention [14]. Several studies have identified that contact and support from clinicians or peers in the form of telephone, email, Web-based forums, or e-chats can help improve adherence to mobile and Internet-based interventions; people with psychosis may particularly benefit from this support [17,18]. Mohr et al [19] outline a “supportive accountability model” whereby a supportive social presence may positively influence accountability, expectations, and bond during a mobile or Web-based intervention. This predictor has some credibility as Day et al [20] found that for acute inpatients with psychosis, a positive relationship with a clinician was related to adherence to medication and a positive attitude toward treatment. In addition, LeClerc et al [11] established that a good therapeutic alliance improved adherence to psychosocial treatment. This review conducted a preliminary examination of the level of social presence and support that is offered in each intervention.

The second potential predictor of adherence is the level of service user involvement in the development of the intervention. This has been highlighted as vital for effectiveness and adherence to interventions [21]. The sense of involvement in the project may promote self-efficacy and therefore accountability to the intervention [19]. Recently, Wykes and Brown [21] emphasized the importance of providing service users with choice, for example, the choice of digital or face-to-face interventions, or a combination of the modalities [22]. Choice leads to a greater feeling of control; this may tap into intrinsic motivation that is important for adherence to interventions [19]. This review highlights any studies that involve service users in the development and improvement of the interventions and the potential impact on adherence. This review updates Alvarez-Jimenz et al’s [14] findings; we examined rates of adherence to mobile or Internet-based interventions, trials, or observational studies for people with psychosis.

## Methods

This systematic review was conducted following the preferred reporting items for systematic reviews and meta-analyses (PRISMA) guidelines and recommendations for conducting and reporting systematic reviews (see Multimedia Appendix 1) [23].

### Eligibility Criteria

The following PICOS criteria [24] were adopted for study inclusion: (1) population: adults (18-65 years); at least 75% of participants have a diagnosis of schizophrenia spectrum disorder according to diagnostic and statistical manual of mental disorders (DSM)-IV or the international classification of diseases (ICD)-10; (2) interventions, trials, or observational studies involving Web-based, mobile, e-technology or Web-based interfaces enabling peer-to-peer contact, patient-to-expert communication, or interactive psycho education or therapy; flexible, accessible monitoring, self-help, and symptom management; (3) comparison group: none were specified; (4) outcomes: at least one measure of adherence or dropout; and (5) study design: as this study aimed to provide an overview of the current state of the field, generous inclusion criteria were adopted. Types of studies included all primary group studies including RTCs; cross-sectional, longitudinal, and comparison studies with and without a control group; cross-over trials, case controls or cohort studies; observational studies with experience sampling components (ESM); and feasibility or acceptability studies. The following exclusion criteria were included: (1) publications written in a language other than English, (2) conference abstracts and theses not published in a peer-reviewed journal, and (3) book chapters.

### Information Sources and Search Strategy

The following databases were systematically searched from August 2013 to November 2016: OVID including MedLine, EMBASE and PsychInfo, Pubmed and Web of Science. The following terms were used in the keyword search of abstracts and titles: (Internet or online or Web-based or website or mobile) AND (bipolar disorder or manic depression or manic depressive illness or manic-depressive psychosis or psychosis or schizophr* or psychotic). Additionally, hand-searching was performed on 5 key journals (Schizophrenia Bulletin, Schizophrenia Research, Journal of Medical Internet Research, Telemedicine and E-health, and Psychiatric Services) along with the reference lists of included primary studies. The term “adherence” was purposely not included in the search terms as most studies do not include references to reported adherence in the title or abstract [16].

**Figure 1:**
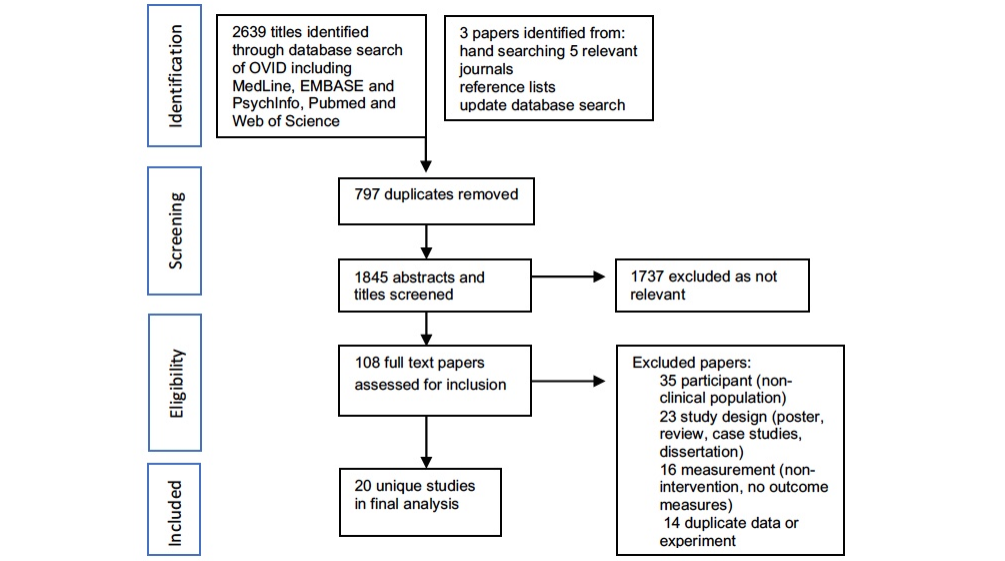
Preferred reporting items for systematic reviews and meta-analyses (PRISMA) flowchart.

### Study Selection

Titles and abstracts of articles were scanned independently by 2 researchers (CK and ZH). Articles deemed potentially eligible were retrieved in full and independently reviewed (CK and ZH). Disagreement between researchers was dealt with by consensus with a senior member of the research team (TW).

### Data Collection Process

A standard form was used to extract data from selected studies to create 7 results tables. Tables 2-7 comprise: (1) randomized intervention studies, (2) feasibility or acceptability studies, and (3) observational studies. Tables 2-4 include the following study characteristics: study source, sample size, gender, age, diagnosis, study design, purpose of intervention, and control group. Tables 5-7 include characteristics of interventions: levels of adherence, dropout, type of social presence, service user involvement, and measurement of participant feedback.

### Assessment of Methodological Quality and Procedures

The study quality was assessed separately for RCTs, feasibility studies, and observational studies (nonrandomized studies). The RCTs and feasibility studies were separately assessed using the clinical trials assessment measure (CTAM) [25]. The CTAM was designed to assess trial quality specifically in trials of psychological interventions for mental health. It contains 15 items grouped into six areas that are important for assessing bias in psychological interventions that include sample size, recruitment method, allocation to treatment, assessment of outcome, control groups, and description of treatments and analysis. Each study is rated out of a total of 100. This scale has good interrater reliability (.96) and high concurrent validity (.97) [26]. Observational studies were assessed using the Downs and Black scale [27]. This scale comprises 27 questions assessing key areas of methodological quality for nonrandomized studies for systematic reviews. It includes questions on reporting, external validity, bias, confounding, and power. This scale was modified slightly for this study. The question on power (see question 27 of the scale) was simplified to a rating of 1 or 0 following the practice in other reviews [2,28]. Each study is rated out of a total of 28 points. Scores are classified in the following ranges: excellent 26-28, good 20-25, fair 15-19, and poor less than 15. Two reviewers (CK and ZH) independently assessed the quality for all of the included studies. All of the first authors of the included articles were contacted to approve their CTAM or Downs and Black rating and if necessary provide further information to ensure that the quality of the study was not confused with the quality of the reporting.

## Results

### Study Selection

The search strategy returned 2639 titles and abstracts. After removal of 797 duplicates, 1842 titles and abstracts were screened and 108 full text papers were assessed for inclusion. In total, 20 studies met the inclusion criteria (see summary in Figure 1; PRISMA flowchart).

### Study Characteristics

Study characteristics are summarized in Tables 2-4. Six were randomized controlled interventions; 7 were feasibility, acceptability studies; and 7 were observational studies. In total, 656 participants with a diagnosis of schizophrenia spectrum disorders and a mean age ranging from 20-48 years participated. Sixteen studies included individuals with schizophrenia or schizo-affective disorder, 1 study included people with first episode psychosis, 1 included individuals with a dual diagnosis of schizophrenia and substance misuse, and 2 included people with nonaffective psychosis.

**Table 2:** Study characteristics: randomized controlled trials with pre and post outcomes and control group.

First author and year	Study source (country)	n (%) (male)	Age Mean (SD)	Specific diagnosis (eg, FEP^a^, chronic)	Study design	Description of study and type of technology	Control group	Outcome measures
Palmier-Claus 2013 [29] (also reported in Ainsworth et al, 2013) [30]	United Kingdom	24 (19)	33.04 (9.5)	Nonaffective psychosis	Randomized repeated measure cross-over design	Use of mobile phone or text messaging for real time assessment of symptoms	Cross-over control group	Qualitative interviews to assess perceptions and experiences of devices, PANSS, quantitative feedback questionnaire
Van der Krieke 2013 [31]	Netherlands	73 (39)	Intervention: 37 (12.35), control: 40 (13.47)	Nonaffective psychosis, DSM^c^criteria	Randomized controlled trial	Web-based information and decision tool to help patients identify needs and treatment options	TAU^d^	Patient-rated COMRADE^e^CSQ^f^
Kurtz 2015 [32]	United States	56 (41)	COG REM^g^group: 36.1 (12.8), control: 37.1 (12.1)	Schizophrenia or schizoaffective disorder	Randomized treatment trial, quasi experimental design, blinded raters	Social skills training combined with Web-based cognitive training (COG REM) to improve memory and attention	TAU and social skills training combined with computer skills training instead of COG REM training	Neurocognitive assessment, WAIS^h^, and others, social skills performance assessment, and quality of life scale
Smith 2015 [33]	United States	32 (17)	Intervention: 40.8 (12.2) Control: 39.1 (10.6)	Schizophrenia and schizoaffective disorder	Randomized control study, blinded raters	Efficacy of virtual reality job interview training on job outcomes and confidence	Waitlist controls	Posttest video role plays of interviews scored by blinded raters, self-report interviewing confidence, 6-month follow-up data on employment outcome
Beebe 2014 [34]	United States	30 (11)	48.7 (11.6)	Schizophrenia spectrum disorders	Small randomized control study	Comparing the effect of telephone calls only, text message only, and telephone calls and text messages on symptoms and medication adherence	Cross-over groups	Symptoms: BPRS^i^, medication adherence scores
Moritz 2016 [35]	Germany and United Kindom	58 (27)	Intervention: 38.9 (11.78), Waitlist controls: 43.41 (8.42)	Schizophrenia	Small randomized control study	Examined whether a Web-based intervention for depression can ameliorate depressive symptoms in schizophrenia	Waitlist controls	CES-D^j^depression scale, PHQ-9^k^, paranoia checklist, PANSS

^a^FEP: first episode psychosis.

^b^PANSS: positive and negative syndrome scale.

^c^DSM: diagnostic and statistical manual of mental disorders.

^d^TAU: treatments as usual.

^e^COMRADE: combined outcome measure for risk communication and treatment decision making effectiveness.

^f^CSQ: client satisfaction questionnaire.

^g^COG REM: cognitive remediation.

^h^WAIS: Wechsler adult intelligence scale.

^i^BPRS: brief psychiatric rating scale.

^j^CES-D: Center for Epidemiologic Studies Depression.

^k^PHQ-9: patient health questionnaire- 9.

**Table 3:** Study characteristics: feasibility studies.

First author and year	Study source (country)	n (%) (male)	Age	Specific diagnosis (eg, FEP^a^, chronic)	Study design	Description of study and type of technology	Control group	Outcome measures
Nahum 2014, [36]	United States	34 (25)	Schizophrenia: 23.8 (3.2), control: 23.6 (3.6)	Schizophrenia spectrum disorder	Case-control study	Feasibility of use and efficacy of a novel neuroplasticity based Web-based training program (SocialVille)	Yes, matched healthy controls	Measures of attrition, compliance, and social cognition; facial memory, emotional prosody identification, emotion, and social perception, Functioning, QoL^b^, social and role scales
Gleeson 2014 (update of Alvarez-Jimenez, 2013) [37]	Australia	20 (10)	Mean 20.3	FEP	Single group design	Safety of HORYZONS Web-based psychosocial Internet-based intervention, including peer-to-peer networking, psychoeducation, Web-based psychosocial intervention modules	No	SCID^c^, BPRS^d^, CDSS^e^, BAI^f^, Feasibility; usage of Web-based system, User experience questionnaire, safety
Ben-Zeev 2014 [3]	United States	17 (10)	Mean 40.47	Dual diagnosis schizophrenia and schizoaffective disorder and substance misuse	Single group design	Feasibility study, clinical social worker sent daily text messages to assess medication and clinical status	No	Usability and satisfaction questionnaire, working alliance inventory
Ben-Zeev 2014, [38]	United States	33 (20)	45.9 (8.78)	Schizophrenia or schizoaffective disorder	Single group design	Feasibility of mobile app resources to facilitate real time illness self-management; mood regulation, medication management, social functioning, sleep, participants asked to complete assessment then intervention if required 3x daily	No	PANSS^g^, BDI^h^, BMQ^i^, acceptability or usability measure, correlation between symptoms and use of phone
Palmier-Claus 2013 (see Palmier-Claus et al, 2012 for main study), [39]	United Kingdom	44 (28)	Acute: 36.8 (10), remitted: 35.5 (8), and UHR: 22 (4.4)	Acute schizophrenia and remitted, UHR	3 groups of patients with different levels of psychosis	Feasibility of a mobile phone based momentary assessment in individuals with psychosis for clinical management and research purposes	none	Calgary depression scale, momentary assessment scales, PANSS
Ventura 2013 [5]	United States	9	Not applicable	Schizophrenia, clinically stable	Pilot single group design	Acceptability of PositScience’s Internet-based brain fitness program using auditory discrimination tasks	None	MATRICS neuro-cognition, clinical global impression of cognition in Schizophrenia, brief questionnaire on knowledge of cognition, outcome rating scale
Schlosser 2016 [40]	United States	20 (17)	Stage 1: 23.40 (2.6), stage 2: 23.3 (3.7)	Schizophrenia spectrum disorders	Pilot single group design	Feasibility and acceptability of implementing PRIME^j^, a mobile app intervention	None	Feasibility measures, adherence measures, satisfaction questionnaires

^a^FEP: first episode psychosis.

^b^QoL: quality of life.

^c^SCID: structured clinical interview for DSM disorders.

^d^BPRS: brief psychiatric rating scale.

^e^CDSS: Center for Doctoral Studies in social and behavioral sciences.

^f^BAI: Beck Anxiety Inventory

^g^PANSS: positive and negative syndrome scale.

^h^BDI: Beck depression inventory.

^i^BMQ: beliefs about medicines questionnaire.

^j^PRIME: personalized real-time intervention for motivation enhancement.

**Table 4:** Study characteristics: observational and experience sampling method studies.

First author and year	Study source (country)	n (%) (male)	Age Mean (SD^a^)	Specific diagnosis (eg, FEP^b^, chronic)	Study design	Description of study and type of technology	Control group	Outcome measures
Brenner 2014 [22]	United States	24 (17)	44.88 (9.27) years	Schizophrenia or schizoaffective disorder	Single group design	Hand-held device to prompt in the moment ratings of positive and negative affect	No	Comparison of baseline scores and momentary affective forecasting throughout the week
Kimhy 2014 [17]	United States	104 (55)	Schizophrenia: 32.15 (9.19) years, control: 23.95 (5.01)	Schizophrenia spectrum disorder	Case-control study	Rating of momentary emotions (sadness, anxiety, anger, and happiness) using mobile electronic devices	Yes, healthy controls	Measures of emotional granularity from ESM^c^responses and social functioning: PSRS^d^, interview, ability task (MSCEIT^e^) Toronto Alexithymia scale, or difficulty identifying feelings or test of reading ability; WTAR^f^, BAI^g^, BDI^h^, symptoms; SAPS^i^, Neurcog; MATRICS
Hartley 2014 [41]	United Kingdom	32 ( 22)	33 (10.7) years	Schizophrenia spectrum disorders, 3+ on the PANSS^j^for hallucinations	Single group design	ESM using a palm computer to capture whether worry and rumination are associated with persecutory delusions and hallucinations	None	Metacognitions around worry; Negative beliefs about ruminations scale, meta-worry questionnaire,
Kimhy 2014 [9]	United States	33 (18)	27.8 (6.3) years	Schizophrenia spectrum disorders, in patient setting	Single group design	The use of mobile devices to monitor symptoms in in-patient environments	None	Self-report rating of mood and symptoms
So 2013 [42]	China and the United Kingdom	26 (13)	36.12 (range 20-63) years	In-patients with acute delusions scoring 4+ on the PANSS, schizophrenia spectrum disorder	Single group design	The use of mobile devices (PDA) to monitor symptoms in inpatient environments after the introduction or reintroduction of antipsychotic medication	None	Symptoms: SAPS, PANSS, PSYRATS^k^
Sanchez 2014 [43]	United States	88 (61)	Schizophrenia: 39.55 (13.95), control: 36.83 (14.89)	Schizophrenia and Schizoaffective disorder	Case-control study	Ecological momentary sampling to examine the relationship between emotion, experience, and environment	Healthy control group	PANSS, MATRICS neurocognitive battery
Ben-Zeev 2016 [6]	United States	20 (16)	39 (12)	Schizophrenia spectrum disorders	Pilot single group design	Acceptability of mobile behavioral sensing	None	Usability and acceptability measures

^a^SD: standard deviation.

^b^FEP: first episode psychosis.

^c^ESM: experience sampling method.

^d^PSRS: positive symptom rating scale.

^e^MSCEIT: Mayer-Salovey-Caruso emotional intelligence test.

^f^WTAR: Wechsler test of adult reading.

^g^BAI: Beck Anxiety Inventory

^j^BDi: Beck depression inventory.

^i^SAPS: scale for the assessment for positive symptoms.

^j^PANSS: positive and negative syndrome scale.

^k^PSYRATS: psychotic symptom rating scales.

**Table 5:** Characteristics of interventions and rates of adherence: randomized controlled trials with pre and post outcomes and control group.

First author and year	Length of study	Adherence measure and rate	Dropout rate (%)	Type of social presence	Frequency of social presence	Service user involvement in development	Measure of participant feedback and rating of acceptability
Palmier-Claus 2013 (also reported in Ainsworth et al, 2013) [29,30]	4x a day for 6 days	% of participants completing the intervention: 88, (across all participants)	1 asked to have SMS^a^stopped 2 days early due to rumination (4.1%)	Once or twice per week based on participants preference	Once or twice per week based on participants preference	Participants were interviewed about their experience	Qualitative interviews with range of perspectives on usability, all participants completed the feedback assessments
Van der Krieke 2013 [31]	6 weeks, self-directed use of website	% of participants completing the intervention: 71% used full functionality of the website	40(55%)	Assist was available to answer questions over the phone anytime	3 days a week	Open interviews with 15 patients to evaluate the intervention	30 used the Web program
Kurtz 2015 [32]	COG REM^b^treatment: 50 min/day 3 days/week for 23 weeks SST^c^: 50 min/day, two days/week, for 23 weeks Computer skills: Target 50 hours over 23 weeks	% of participants completing the intervention: 100%, (min criteria for inclusion; all individuals received at least one session)	8(14.28%)	Interaction with clinician for both COG REM and computer Skills training groups SST group: 2x per week for 50 min, led by researchers	Not applicable	Not applicable	SST Mean number of sessions=32.3 COG REM Mean number of sessions=31.9 Computer skills=Mean number of sessions=32.2
Smith 2015 [33]	Up to 10 hours of virtual interviews over the course of 5 visits	Mean % of entries completed: 90% of sessions attended and completed	2(6%)	Basic contact during computer intervention	During intervention only briefly	None reported	90% attendance rates of sessions
Beebe 2014 [34]	3 months	Mean % of entries completed: 81.60 (across all participants)	2(6.6%)	Various: weekly telephone calls, daily text messages, both	Various	None reported	Phone calls plus text message group higher adherence by a mean of 5.3%
Mortiz 2016 [35]	3 months	% of participants completing the intervention; 28% used it once a week	9(15%)	None- unguided	None	None reported	Feedback on use of the program, 72% rated the quality of the program as good to excellent

^a^SMS: short message service.

^b^COG REM: cognitive remediation.

^c^SST: social skills training.

**Table 6:** Characteristics of interventions and rates of adherence: feasibility studies.

First author and year	Length of intervention	Adherence measure and rate	Dropout rate (%)	Type of social presence	Frequency of social presence	Service user involvement in development	Measure of participant feedback and rating of acceptability
Nahum 2014 [36]	Total of 24 h of Web-based training, 1-2 h per day for 6-12 weeks	% of participants completing the intervention: 78 (completed 24 h of the intervention across all participants)	8(22-23% attrition rate)	None reported	None reported	Subjects rated their satisfaction in the training program	Subjects took 8.1 weeks (mean) to complete the 24 h of training
Gleeson 2014 (update of Alvarez-Jimenez, 2013) [37]	1 month	% of participants completing the intervention: 60 (completed at least three modules, eg, 33%)	None: all accessed modules	Peer-to-peer Web-based social networking Coaches (expert moderator)	Coaches moderated Web-based activity 2 hours/day weekdays, 1h/day weekend	Developed with service user group	70% completed 30 weeks, 60% completed >3 Web-based therapy modules, and 75% reported a positive experience
Ben-Zeev 2014 [3]	12 weeks	Mean % of entries completed: 87.00 (mean response rate to text messages for all participants)	5 (11%)	Mobile interventionist: clinical social worker	Daily, up to 3 text messages a day	None described	Participants responded to 87% (mean) of messages and 90% rated the intervention easy to use, useful, and fun
Ben-Zeev 2014 [38]	1 month	Mean % of entries completed: 86.5 (rate of access to the system for all participants)	1(3%)	Researcher called participant to check in and assist with technical difficulties	1x/week	Developed through service user feedback	90% rated the intervention as highly acceptable, 12% reported it was a complicated intervention, reductions in symptoms PANSS^a^and BDI^b^
Palmier-Claus 2013 [39]	6x a day for 7 days	Mean % of entries completed:72 for those who were compliant with the intervention (eg, completed 33% of data)	8(18%)	Researcher telephoned participant at least once per week to offer advice and encouragement	Once or twice per week based on participants preference	None described	82% of participants met compliance criteria of completing at least 33% of the entries
Ventura 2013 [5]	6 weeks, 2 hours/week	Mean % of entries completed: 75 (response rate across all participants)	1(11%)	Regular phone contact with the study team	Not applicable	None reported	5 participants completed 12 or more sessions (75% of patients reached adherence criteria)
Schlosser 2016 [40]	At least once per week for 12 weeks	Mean % of entries completed (challenges completed)	0	Ranged from once a week in stage 1, to 5x a week in stage 2	Once a day, modified to the service users preference	User-centered design model where participants gave feedback on the iterative development of PRIME^c^in two stages	Mean overall satisfaction with PRIME 8/ 10

^a^PANSS: positive and negative syndrome scale.

^b^BDI: Beck depression inventory.

^c^PRIME: personalized real-time intervention for motivation enhancement.

**Table 7:** Characteristics of interventions and rates of adherence: observational or experience sampling method studies.

First author and year	Length of intervention	Adherence measure and rate	Dropout rate (%)	Type of social presence	Frequency of social presence	Service user involvement in development	Measure of participant feedback and rating of acceptability
Brenner 2014 [22]	6x a day for 7 days	Mean % of entries completed: 98.10 (response rate across all participants)	None	Researcher called participant to check in and assist with technical difficulties	2x/week	None described	Response rate 98.1%
Kimhy 2014 [17]	10x a day for 2 days	Mean % of entries completed: 79.15 (response rate across all participants)	35(37%)	None reported	None	None described	Not reported
Hartley 2014 [41]	10x a day for 6 days	Mean % of entries completed: 59 (response rate for completers; completion of the schedule defined as completing at least half of the entries (n=27))	5 (15 %)	During the first day, patients contacted to ensure functional equipment	Once in a week, but if needed additional phone contacts were arranged	Feedback questionnaire about involvement	
Kimhy 2014 [9]	10x a day for 1 days	Mean % of entries completed: 81 (response rate for all participants)	1(3%)	Introduction session for 20 min on first day	None reported	None reported	81% response rate
So 2013 [42]	14 days 7x a day, randomly	Mean % of entries completed:70.7 (response rate in participants who completed at least 1/3 of entries)	5 (19%)	Contacted by researcher at least 2x during first week, to offer support and remind to change battery	Participants were encouraged to contact researcher by phone if problems	None reported	16 participants met criteria for minimum compliance, completing 30 or more diary entries
Sanchez 2014 [43]	Phone call 4x a day for 7 days	Mean % of entries completed: 80.16 (response rate for all participants with schizophrenia)	4 (4%)	Participants were called 4x a day	4x a day, each patient was interviewed about their environment, goals, and activities	None reported	Response rate to calls was 80.6% in patients and 81.3% in controls
Ben-Zeev 2016 [6]	Outpatients 2 weeks 12 hours a day, inpatients 1 week 12 hours a day	% of participants completing the study: 95% (one participant did not charge the phone regularly)	0	Once at the beginning to set up phone	Once	Post tudy usability and acceptability questionnaires	95% felt comfortable using the mobile phone sensing system, and 70% understood how it worked and did not have difficulty keeping the device with them

### Quality Assessment

Trial quality assessment scores are summarized in Tables 8 and 9. The mean study quality score for the RCTs on the CTAM was 77.3 (range 62-88). Five [31-35] of the RCT studies were deemed to be of adequate trial quality (rating of 65+), except for Palmier-Claus et al [29], which received ratings of 62. As expected due to the lack of randomization, feasibility studies (n=7) had a lower mean score (44.7). The mean quality rating for the observational studies, was 20 and ranged from 17- 24. Three studies fell into the “good” classification range and 4 were “fair.”

**Table 8:** Clinical trials assessment measure (2004), assessment for randomized controlled trials, and feasibility studies.

Author and year	Total CTAM^a^(max 100)	Sample (Q1,Q2) (max 10)	Allocation (Q3,Q4,Q5) (max 16)	Assessment (Q6,Q7,Q8,Q9,Q10) (max 32)	Control (Q11) (max 16)	Analysis (Q12,Q13) (max 15)	Treatment description (Q14,Q15) (max 11)
Gleeson et al, 2014^b^[37]	44	2,0= 2	0	10,6,0,0,0= 16	0	5,6,4= 15	3,3,5= 11
Ben-Zeev et al, 2014^b^[3]	36	2,0=2	0	10,6,0,0,0= 16	0	5,6,4= 15	3,0,0,= 3
Ben-Zeev et al, 2014^b^[38]	44	2,5=7	0	10,6,0,0,0= 16	0	5,6,4= 15	3,3,0= 6
Nahum et al, 2014^b^[36]	44	2,0= 2	0	10,6,0,0,0=16	0	5,6,4=15	3,3,5=11
Palmier-Claus et al, 2013^b^[39]	39	2,0=2	0	10,6,0,0,0,=16	0	5,6,4=15	3,3,0=6
Palmier-Claus et al, 2013 [29]	62	2,0=2	10,3,0=13	10,6,0,0,0=16	10	5,6,4=15	3,0,3=6
Van der Krieke et al, 2013 [31]	78	2,5=7	10,3,0=13	10,6,10,0,0=26	6	5,6,4=15	3,3,5=11
Ventura et al, 2013^b^[5]	44	2,0=2	0	10,6,0,0,0=16	0	5,6,4=15	3,3,5=11
Kurtz et al, 2015 [32]	88	2,5=7	10,0,3=13	10,6,10,3,3=32	10	5,6,4=15	3,3,5=11
Smith et al, 2015 [33]	79	2,0=2	10,3,0=13	10,6,10,3,3=32	6	5,6,4=15	3,3,5=11
Beebe et al, 2014 [34]	75	2,0=2	10,3,0=13	10,6,10,3,0=29	10	5,6,4=15	3,3,0=6
Mortiz et al, 2016 [35]	82	5,5=10	10,3,3=16	10,6,10,3,0=29	6	5,6,4=15	3,3,0=6
Schlosser et al, 2016^b^[40]	62	2,0=2	10,0,0=10	10,3,10,0,0=23	6	5,6,4=15	3,3,0=6

^a^CTAM: clinical trials assessment measure.

^b^The study is designed as a feasibility or acceptability trial. For ratings of treatment description: Q14 score 3 if website or mobile interface adequately described; for ratings of handling of dropouts, if dropouts described and reasonably analyzed score of 4 given.

**Table 9:** Trial quality characteristics for nonrandomized controlled trials: Downs and Black (1998) ratings.

Checklist Question	Brenner and Ben-Zeev 2014 [22]	Kimhy 2014 [17]	Kimhy 2014 [9]	Hartely 2014 [41]	So 2013 [42]	Sanchez 2014 [43]	Ben-Zeev 2016 [6]
Question 1	1	1	1	1	1	1	1
Question 2	1	1	1	1	1	1	1
Question 3	1	1	1	1	1	1	1
Question 4	1	1	1	1	1	1	1
Question 5	2	2	2	2	2	1	1
Question 6	1	1	1	1	1	1	1
Question 7	1	1	1	1	1	1	1
Question 8	1	1	1	1	1	0	1
Question 9	1	0	1	0	1	0	1
Question 10	0	1	1	1	1	1	1
Question 11	0	UTD^a^	UTD	0	1	1	1
Question 12	0	UTD	1	1	1	0	1
Question 13	1	1	1	1	1	1	1
Question 14	0	0	0	0	0	0	0
Question 15	0	UTD	UTD	UTD	0	UTD	UTD
Question 16	1	1	1	1	1	1	1
Question 17	1	1	1	1	1	1	UTD
Question 18	1	1	1	1	1	1	1
Question 19	1	1	1	1	1	1	UTD
Question 20	1	1	1	1	1	1	1
Question 21	1	1	1	1	1	1	1
Question 22	1	1	1	1	1	UTD	UTD
Question 23	0	0	0	0	0	0	0
Question 24	0	0	0	0	0	0	0
Question 25	0	1	1	1	1	1	UTD
Question 26	1	UTD	UTD	1	1	0	1
Question 27	0	0	UTD	1	1	0	0
Total	19	19	21	22	24	17	18

^a^UTD: unable to determine.

### Adherence: Types of Measurement Across Studies

**Figure 2:**
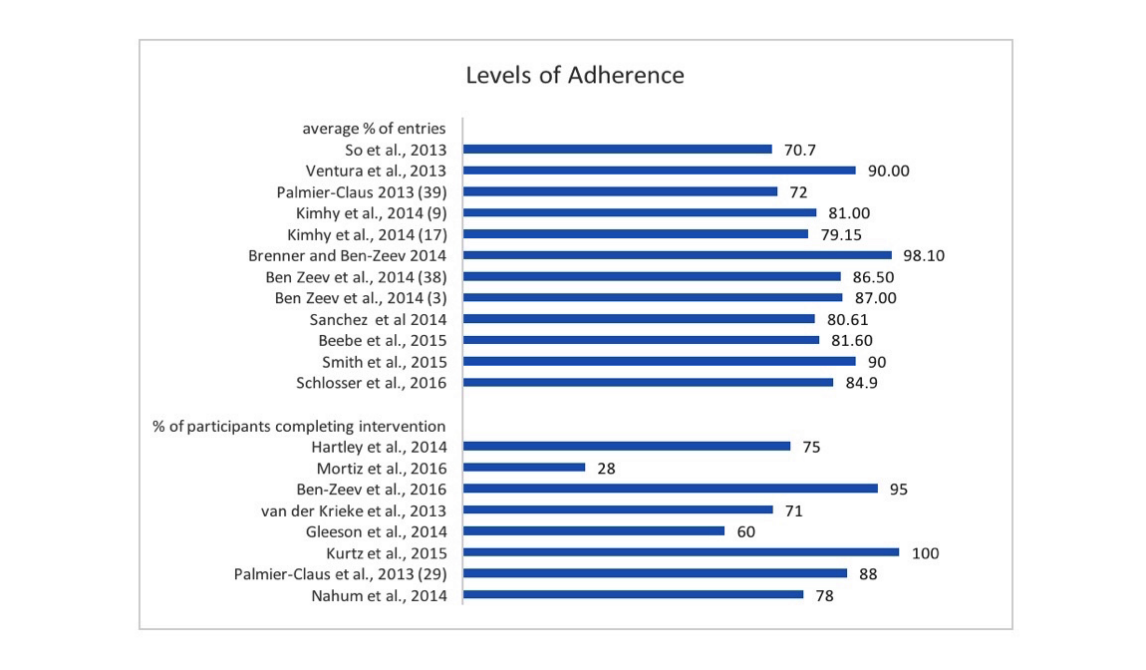
Adherence across all studies: mean percent of entries completed in each study followed by percentage of participants completing the intervention.

The most common measures of adherence were percent of intervention completed by participants and percentage of participants completing the intervention. Figure 2 displays the types of adherence measure used and the level of adherence for each study. For the 12 studies reporting mean % of the intervention completed by participants, adherence ranged from 70.7-98.0% with a mean of 83.4%. For the 8 studies reporting the percentage of participants completing the intervention, adherence ranged from 28- 100% with a mean of 74.3%. All of the studies also listed the number of participants that dropped out of the study. This ranged from 0-55% with a mean of 12.3% dropout across both observational and intervention studies.

#### Approach 1: Analysis of Dropout

See Tables 5-7 for details of rates of dropout. Five studies analyzed the relationship between specific variables and dropout. In terms of the variables of age or gender and dropout, most of the studies found no relationship [29,41,42]; however, Van der Krieke et al [31] found that the dropouts tended to be younger and male. Hartley et al [41] and So et al [42] did not find a relationship between symptom severity and dropout; however, Palmier-Claus et al [29] (also reported in the original study [30]) found that higher severity on the positive and negative syndrome scale’ (PANSS) positive symptom subscale predicted dropout. Finally, Sanchez et al., [43] found that the level of cognitive functioning did not predict completion of the study. See Table 10 for a summary.

**Table 10:** Summary of findings for predictors of dropout and adherence.

Study	Chronicity or duration of symptoms	Cognitive functioning	Severity of symptoms	Age	Gender
Van der Krieke et al, (2013) [31]	Yes^a^			Yes	Yes
Ben Zeev et al, (2014) [38]		No^b^	No		
Palmier-Claus et al, (2013) [39]	No		Yes	No	No
Schlosser et al, 2016 [40]	No		No	No	No
Kimhy et al, (2014) [17]			No		
Hartley et al, (2014) [41]			No	No	No
So et al, (2013) [42]			No	No	No
Sanchez et al, (2014) [43]		No			
	Duration of the study	Time to complete an entry			
Palmier-Claus et al, (2013b) [29]	Yes	No			

^a^“Yes” indicates that the variable was found to significantly predict nonadherence or drop out.

^b^“No” indicates that no relationship was found.

#### Approach 2: Analysis of Within Study Predictors of Adherence

Six studies conducted within-study analyses to examine adherence predictors and found few significant predictors of adherence. Van der Krieke et al [31] analyzed the chronicity of symptoms and reported that service users with first episode psychosis used a Web-based decision aid autonomously more often than service users with chronic psychosis. For those who required assistance from the research team to complete the intervention, 56% were service users in long-term care. However, the report does not provide specific statistical data.

In terms of intervention specific factors, Palmier-Claus et al [29,30] found no relationship between the length of time taken to complete an entry and the number of entries completed by an individual. They also examined number of entries completed across the number of weeks of the study. They found that more entries were completed in the first week than the second week of the intervention and participants rated more highly the question, “were there times when you felt like not answering?” during the second week.

#### Approach 3: Poststudy Questionnaires on Participants’ Perspectives on Adherence

**Figure 3:**
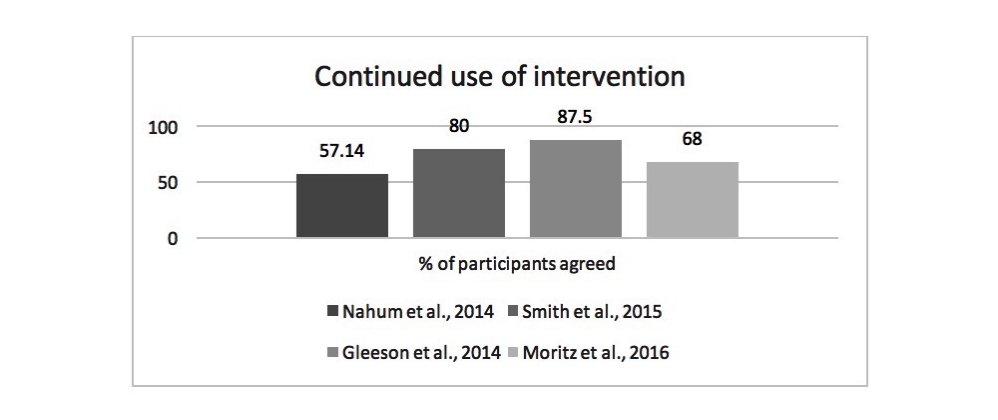
Percent of participants agreed to continued use of intervention.

11 studies retrospectively asked participants to provide questionnaire-based qualitative or quantitative feedback about their experience of the study or intervention. All the studies used different rating scales (eg, Treatment Experience Questionnaire in Smith et al [33], idiosyncratic quantitative feedback questionnaire in Palmier-Claus et al [29], and idiosyncratic SocialVille program rating in Nahum et al [36]) and therefore it is difficult to draw comparisons across studies. Four studies specifically asked whether participants would continue to use the intervention [33,35-37]; see Figure 3). For 4 studies, the mean percent of participants who agreed to continue to use the intervention was 73.1%.

#### Approach 4: Analysis of Specific Intervention Manipulations and Effect on Adherence

Two studies were designed to manipulate conditions that may have an impact on adherence. Palmier-Claus et al [29,30] compared two different types of interventions: SMS text-only (short message service, SMS) interface or a mobile phone–based graphical app. They assessed the acceptability and feasibility of each device and found that participants completed more data points when using the mobile phone interface (mean entries=16.5) compared with the SMS text-only interface (mean entries=13.5; *P*=.002). Schlosser et al [40] increased the frequency and intensity of contact from a research coach from once a week to 5 times a week. This led to improved rates of adherence, for example, number of logins increased from 3.51 days/week to 4.69 days/week.

Interestingly two interventions found that adherence significantly affected the intervention efficacy. Smith et al [33] found that completing more training trials of a virtual reality job interview training correlated with fewer weeks searching before securing a job (*P*<.001) and greater self-confidence (*P*=.03).

Ben-Zeev et al [38] analyzed symptom change throughout the intervention and any related association to adherence and found that change in participants’ Beck Depression Inventory (BDI) scores were significantly correlated with use of mobile intervention; less frequent use of the FOCUS mobile intervention was associated with a the greater the reduction in depression score. Change in PANSS scores was not associated with use of the FOCUS app.

### New Potential Predictors of Adherence

#### Potential Predictor: Social Presence Analysis

To assess Mohr et al’s [19] “supportive accountability” model (social presence leads to better adherence), we examined the amount of contact for each study and the level of adherence to the intervention. As there is heterogeneity across the studies, we provide a narrative synthesis. Across all 20 studies the mean number of contacts per week from a researcher or clinician was 4.4 and it ranged from 0-28 contacts per week. This included face-to-face, mobile, Web-based or telephone-based contacts.

**Figure 4:**
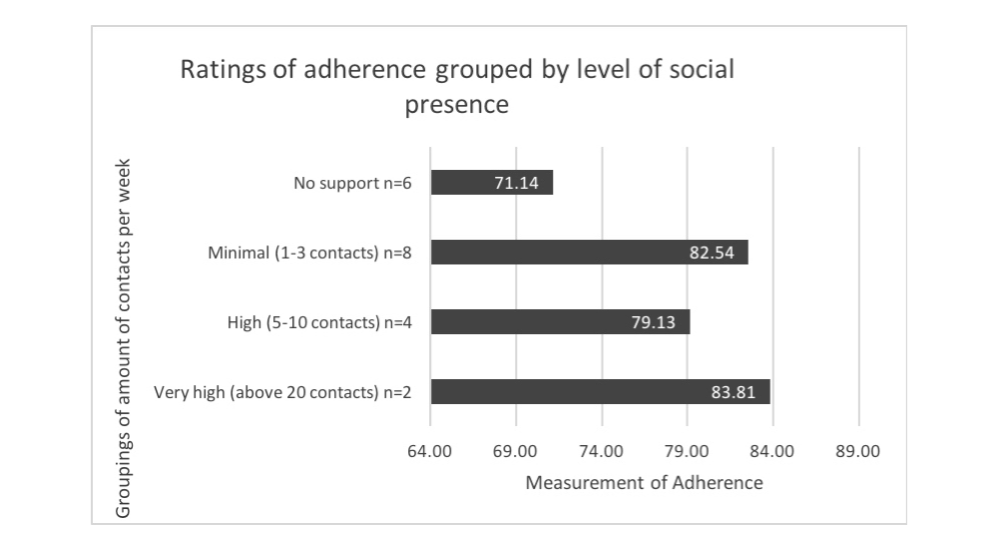
Relationship between social presence and adherence, adherence rates are grouped by frequency of social contact per week from “very high” (20 or more contacts per week), “high” (5 to 10 contacts per week), “minimal” (1 to 3 contacts per week), or “no support” (no contact).

As presented in Figure 4, regardless of level of support there is still a moderate to high rate of adherence across all 20 studies. Interestingly, it appears that studies with very high contact have almost 10% higher rates of adherence (83.8%) than those with no support (71.1%), but studies with minimal contact also had high adherence ratings (82.5%). Anecdotally, the importance of social presence is confirmed from participant reports. Gleeson et al [37] found that 90% of participants cited the use of a Web-based facilitator contributed to their sense of safety when using the Web-based program. All participants either agreed or strongly agreed with statements such as they always felt supported by the Web-based facilitator and 60% reported an increase in feelings of social connectedness. Recently, Schlosser et al [40] found that increasing the frequency of contact with a research coach increased use of the mobile app PRIME significantly. They found that when service users were able to tailor the amount of social support they received, they engaged more with the app.

#### Potential Predictor: Service User Involvement

Of the 20 studies included, only 3 described service user involvement in terms of the development or initial piloting of the intervention.

Coproduction, meaning the collaboration of service users and researchers, in the beginning phases of intervention development has a potential influence on participants’ perception and adherence to the intervention. Ben-Zeev et al [38] used feedback and recommendations from a pilot with service users to develop a mobile intervention, FOCUS, to facilitate real-time mobile illness self-management. They found that participants rated the intervention highly with 90% acceptability and the mean percent of entries completed was 86.5%. Gleeson et al’s [37] HORYZONs program was developed with a service user focus group. It was found that 95% of participants used the social media component, 60% completed the therapy modules, and 75% reported a positive experience with the program. Schlosser et al [40] used an iterative service user feedback process called user centered design (UCD) process. After using the mobile app for 1 week, service users were consulted by means of in-depth interviews about their experience and identified key areas for improvement. The recommended changes were incorporated into the design of the device and this led to a 2 to 3-fold increase in use of the app in week 2. Service users also rated the app at 8 out of 10 in terms of satisfaction. In this case, service users were directly involved in the design, development, and implementation of the new device. When compared with adherence ratings (mean rate of adherence across studies that used different types of adherence ratings) to feasibility studies or RCTs that did not involve service users (mean adherence rate of 78%), service user involvement was associated with higher adherence (mean of 89%), though this is a small number of studies (n=3).

#### Additional Potential Predictor: Duration of Study

**Figure 5:**
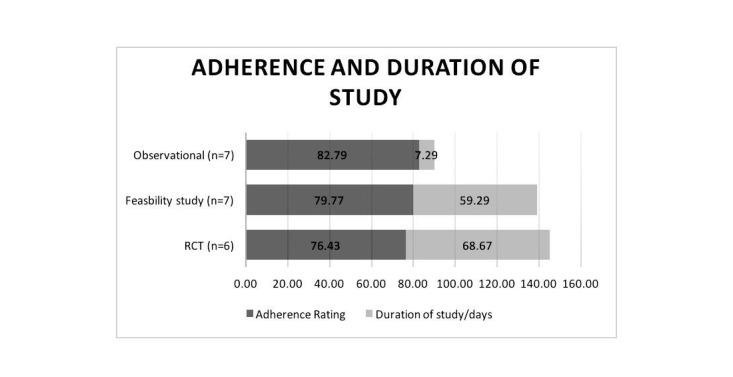
Adherence ratings and the mean duration of the study (number of days) grouped by study type.

Interestingly, a comparison of the duration of the study (number of days participants are expected to be active in the study) and levels of adherence (averaged across both types of adherence ratings) revealed that the studies with the shortest duration had the highest mean rates of adherence (see Figure 5). The duration of the ESM-based studies ranged from 1 day to 14 days and the mean rate of adherence for these studies was 82.7%. Conversely, the duration of the RCT studies ranged from 6-161 days with a mean adherence rating of 76.4%; the duration of feasibility studies ranged from 7 -84 days with a mean adherence rating of 79.7%.

## Discussion

### Principal Findings

This is the first review to document rates of adherence and to explore predictors of adherence to mobile and Web-based interventions for people with psychosis. Overall, from the examination of the four approaches to studying adherence across these diverse studies, we conclude that adherence to mobile and Web-based interventions is not necessarily predicted by service user specific factors such as age, symptoms, or gender. However, people with FEP may prefer an intervention that they can independently access [31]. Additionally, adherence is moderate to high across specific intervention factors such as amount of time to complete an entry and across different study designs. However, service users may prefer the mobile phone interface and may adhere more in the first week of an intervention [29]. This review has important implications for the acceptability and use of current interventions and the development of new ones. For example, offering service users choice in terms of the duration of the intervention and also the mode of delivery may have an important influence on adherence. Some service users may prefer a mobile app whereas others prefer a Web-based platform. Two potential new predictors of adherence were explored: (1) more frequent social support and (2) service user involvement in the intervention development. Providing service users with more input and control may add to the value and use of these interventions.

### The Measurement of Adherence

Overall, adherence rates (whether measuring mean percentage of the intervention completed or percent of participants that complete the intervention) to mobile and Web-based interventions for people with psychosis are in line with adherence rates for similar technology-based interventions for other mental health disorders. Rates of adherence to interventions for depression and anxiety are approximately 66% for self-care interventions [16], and a median 56% for a computerized cognitive behavioral therapy (CBT) intervention [44]. Rates for completion of a Web-based site for personality disorder ranged from 80-100% completion; social phobia reported 70-90% completion and the only post-traumatic stress disorder (PTSD) intervention reported completing rate of 64%. In terms of adherence across different types of interventions (eg, face-to-face; medication-based interventions), completion rates of a one-to-one CBT intervention for psychosis was 55% [45] and 68.3% for a one-to-one CBT intervention for FEP [46]. Overall, the current review found moderate to high levels of adherence to Web-based or mobile interventions for psychosis with a range of 60-100% and a mean of 83%.

In terms of the four approaches to studying adherence, the studies in this review varied in terms of the within-study predictors that are associated with adherence, questionnaires used to assess participants’ perspectives on factors impacting adherence, and whether or not they conducted any experimental manipulations to impact on adherence.

### Predictors of Adherence and Dropout

Only 2 studies found specific predictors of adherence: less chronic symptoms [31] and a higher rate of adherence was found in the first intervention week than the second [29,30]. Although other predictors of adherence were examined (age, gender, cognition, negative symptoms, and persecutory delusions), none were found to have a significant effect. Two studies also found significant predictors of dropout: severity of symptoms [39], younger age, and male gender [31].

Complex analyses, such as the multiple regression analysis performed by Palmier-Claus et al [39] of specific predictors such as service-user factors (symptoms, socioeconomic factors, interpersonal factors, and cognitive factors) along with e-mental health intervention factors (complexity of the interface, cost, and access) should be a priority for future studies. This will inform which service-user group may adhere to different type of interventions.

One interesting area of future research would be to examine the duration, frequency, and intensity of the intervention and the effect that this may have on adherence. Studies that last for several months may have more variable adherence than those that last only 1 week. Additionally, longer adherence is not always synonymous with better outcomes. Palmier-Claus et al [29] found that the longer participants used the app, the greater the increase in their depression symptoms. This has important implications for future research; it could be that people will stop using the app as they improve and should therefore be given the opportunity to stop using the app when they have exceeded the benefit. Ultimately, it may be most effective to allow service users choice of the duration, frequency, or intensity of interventions. With supportive guidance, service users may best be able to decide whether or not a technology is helpful and supportive in their recovery.

### Poststudy Questionnaires

Several studies used participant feedback questionnaires, however, they were all different; some were previously published but most were idiosyncratic and this variability also hindered comparison. A standard questionnaire specifically for Web-based and mobile interventions could provide detailed and comparable information on the service user perspective and experience. Additionally, more independent data collection, perhaps from service user researchers not associated with the study, may provide a more unbiased and critical view of the interventions (eg, [47]). The use of posttrial feedback should be a priority for future research studies.

### Experimental Manipulation

Only 2 studies specifically manipulated variables in an attempt to influence adherence or use of the intervention. Both successfully improved adherence to the intervention (eg, mobile phone rather than text message based delivery; higher frequency supportive contact). Experimental manipulation of variables is vital particularly in terms of the types of technologies service users would prefer, the content of interventions and the level of independence, or clinician involvement in use of the intervention.

### New Predictors of Adherence

“Support” in this review was defined liberally as any type of contact with a clinician or researcher involved in the study. Of the 20 studies, 14 reported some level of clinician or researcher contact. This ranged from very limited initial interaction with a researcher to multiple daily support calls from a dedicated mobile interventionist. It should be noted that 7 of the studies were designed as observation studies with ESM components. In this case, researcher or clinician contact may only occur if service users stop filling in the data. Additionally, ESM studies are usually very short so there is less time for absolute dropout. As evidenced by our comparison with adherence ratings grouped by the duration of the study, ESM studies tended to be the shortest studies with the highest adherence ratings.

At present, it is difficult to draw clear conclusions about the importance of support, as only 2 studies specifically reported data on the effect of the Web-based interventionists [37]. However, as demonstrated by Schlosser et al [40], when the amount of coaching support was increased during the second half of the intervention, it led to increased engagement. In the future, it would be interesting for studies to experimentally manipulate the level of support and then measure the impact on adherence, or correlate the ratings of therapeutic alliance in the intervention and the level of adherence. This will clarify the impact of social presence.

Alvarez-Jimenez et al [14] and Wykes and Brown [21] recommend that service user involvement in intervention development might be an important predictor of adherence. However, in the current dataset, only 3 studies included service users in the development of the intervention, so it is difficult to draw conclusions about the impact on adherence. However, adherence to these interventions was very high (84.9%, 86.5%, and 95%). This is an important area requiring future study.

### Quality of Studies

As expected, the RCT studies were rated more highly (77.3%) than feasibility studies (44.7%). All of the studies had interventions carried out by independent assessors and had adequate handling and assessment of dropouts. Only 4 studies had outcome assessments conducted by assessors blind to group allocation. In terms of observational studies, these studies were classified as either fair or good in terms of the quality. Few studies (n=4) used a method of blind rating of outcomes. This is particularly important when assessing service user satisfaction with the intervention, as researcher involvement may unintentionally bias the ratings. Finally, it is difficult to compare study quality across feasibility, RCT, and observational studies. Currently there is no measure to assess the quality of feasibility studies. The CTAM and Downs and Black scales provide a useful reference point; however, direct comparisons are not possible. In the future, RCTs should be developed from the feasibility studies discussed here, to provide further, high quality support for these initial findings.

### Strengths and Limitations of the Review and Recommendations

One of the main limitations of this study is the difficulty of comparing rates of adherence across studies with different interventions and different outcomes. Although most studies provided data either as percent of individuals completing an intervention or the mean percentage of an intervention completed, these two measures may not provide as accurate information when directly combined. A universal measure of adherence should be adopted in addition to more detailed information on the quantity or quality of adherence. For example, Simco et al [16] recommended including not just the percentage of an intervention completed but the number of exercises per week or log-ins per week to get a more qualitative perspective on use. Along these lines, it will be important for future reviews to separate and compare the modes of delivery in their analysis of baseline adherence levels. For example, the baseline rate of adherence to a mobile phone intervention may be different than for a Web-based intervention; comparisons across and within modes of delivery may provide insight into the types of technologies that are preferred. Finally, it will be important for future reviews to carefully document and unpick any potential risks of harm that service users may experience when using these remote technologies. Reviews should provide an unbiased account of both the benefits and disadvantages of remote interventions, for example, as highlighted by the finding by Ben-Zeev et al (2014b) that participants’ BDI scores were significantly correlated with use of mobile intervention; less frequent use of the FOCUS mobile intervention was associated with a greater reduction in depression score. This is an important finding that should guide further use of this intervention (eg, Ben-Zeev et al, 2016). Any potential negative effects should be carefully explored and documented.

The review provides a comprehensive, up-to-date review of adherence across a variety of intervention types and platforms. The strengths include assessing a broad range of different novel technological interventions from text message-based to Web-based to virtual reality-based programs. This allowed us to demonstrate that adherence across different types of studies and a diverse range of interventions is moderate to high. Although the choice between face-to-face and remote intervention was not examined, this result at least demonstrates potential clinical utility. This review is timely as we included up-to-date literature from the past 3 years to ensure that the reader is informed of the most recent developments. The review also provides an innovative exploration of theoretically proposed predictors of adherence. This is the first review of its kind to explore the importance of service user involvement and support in facilitating adherence.

We conclude that specific service user factors such as age or symptom severity may not have a significant influence on adherence; however, the experience of the service user in terms of the development of these technologies and interventions may be an important factor that requires care and consideration.

## Appendix

Multimedia Appendix 1 PRISMA checklist.

## Abbreviations

CBT: cognitive behavioral therapy
CTAM: clinical trials assessment measure
DSM-5: Diagnostic and Statistical Manual of Mental Disorders
ESM: experiential sampling method
FEP: first episode psychosis
ICD-10: International Classification of Diseases
PRISMA: preferred reporting items for systematic reviews and meta-analyses
RCT: randomized controlled trial
